# Real-world pharmacovigilance study of drug-induced diabetes insipidus from the FAERS database

**DOI:** 10.1530/EC-25-0734

**Published:** 2026-01-02

**Authors:** Hui Lv, Yue Wu, Lan Lan, Nonger Shen

**Affiliations:** Center for Clinical Pharmacy, Cancer Center, Department of Pharmacy, Zhejiang Provincial People’s Hospital (Affiliated People’s Hospital), Hangzhou Medical College, Hangzhou, Zhejiang, China

**Keywords:** diabetes insipidus, drug-induced, FAERS, pharmacovigilance study, disproportional analysis

## Abstract

**Objective:**

This study aims to identify potential drugs associated with diabetes insipidus (DI) and track its epidemiological characteristics using the FDA Adverse Event Reporting System (FAERS) database.

**Methods:**

A retrospective pharmacovigilance analysis was conducted on FAERS data from Q1 2004 to Q4 2024. Disproportionality analyses were performed using the reporting odds ratio and the Bayesian confidence propagation neural network (BCPNN).

**Results:**

A total of 2,189 cases of DI were recorded in FAERS, with a median age of 47.0 years (interquartile range (IQR) 27.0–60.0). Disproportionality analysis identified 71 drugs with positive signals, in which nervous system agents (22 drugs, 31.0%), antineoplastic agents (15 drugs, 21.1%), and systemic anti-infectives (14 drugs, 19.7%) constituted the top three drug classes. Lithium (*n* = 114, information component at the 95% lower credibility interval (IC_025_) = 6.12) and dexmedetomidine (*n* = 105, IC_025_ = 6.79) were identified as the most frequently reported drugs and showed the strongest association with DI. Several drugs, such as aripiprazole, letrozole, tigecycline, and dapagliflozin, were found to have unexpected potential associations with DI.

**Conclusion:**

This study provides a comprehensive overview of drug-induced DI based on real-world data. It highlights the importance of monitoring patients for DI when using certain medications, particularly high-risk nervous system drugs and antineoplastic agents.

## Introduction

Diabetes insipidus (DI) is a clinical syndrome characterized by polyuria (excessive urination exceeding 50 mL/kg body weight/24 h) and polydipsia (excessive drinking greater than 3 liters per day) (1, 2). The disorder arises from impaired regulation of water homeostasis, with two primary subtypes: central diabetes insipidus (CDI), also known as arginine vasopressin (AVP) deficiency, and nephrogenic diabetes insipidus (NDI), referred to as AVP resistance (2, 3). CDI is caused by deficient synthesis and/or secretion of arginine vasopressin from the posterior pituitary gland, and NDI results from renal resistance to AVP. Although congenital forms are present, the majority of cases are acquired, with CDI often associated with trauma, neurosurgical interventions, autoimmune diseases, neoplasia, infections, and idiopathic factors, among other etiologies (4, 5). In contrast, acquired NDI is primarily attributed to multiple underlying causes, including electrolyte abnormalities, hematological disorders, and kidney diseases. In children, CDI is more frequently observed than NDI and is predominantly acquired, whereas NDI is rare and largely hereditary (6, 7).

Drugs have been identified as a significant yet understudied etiological factor in the development of DI (4, 5). Historically, drug-induced DI has been predominantly classified as NDI, with lithium being the most prototypical agent (8). Other drugs associated with DI include antiviral agents (e.g., foscarnet and cidofovir), antibiotics (e.g., demeclocycline), and specific antipsychotic medications (5, 8). However, emerging evidence suggests that certain drugs, particularly immune checkpoint inhibitors (ICIs), may also induce CDI (9). Despite these clinical observations, current knowledge remains fragmented, relying primarily on case reports and small case series. This evidence gap underscores the necessity for rigorous post-marketing surveillance to establish a comprehensive understanding of drug-associated DI risks.

The management of DI depends on the underlying etiology and specific subtype (4, 5). In the case of drug-induced DI, the primary approach is the discontinuation of the causative agent. However, given the overlap in symptoms with other diseases and the rarity of DI (with an incidence of approximately 1:25,000), timely diagnosis and treatment can be challenging (2, 10). Consequently, a comprehensive understanding of high-risk medications that may induce DI, along with the implementation of appropriate drug monitoring, is crucial for the prompt identification and management of drug-induced DI.

The U.S. Food and Drug Administration Adverse Event Reporting System (FAERS) is a spontaneous reporting system that collates adverse event (AE) reports from across the globe (11). This database plays a pivotal role in post-marketing drug surveillance, enabling the detection of potential safety issues that may not have been apparent during clinical trials. This retrospective study aimed to utilize the FAERS database to identify potential medications associated with DI. The findings of this study are expected to enhance our understanding of this complex condition, inform clinical practice, and contribute to the development of more effective strategies for the prevention and management of drug-induced DI.

## Methods

### Data sources

The FAERS database is publicly accessible at https://fis.fda.gov/extensions/FPD-QDE-FAERS/FPD-QDE-FAERS.html and has been released quarterly since the first quarter of 2004. It consists of seven datasets: demographic and administrative information (DEMO), drug information (DRUG), adverse event information (REAC), drug therapy information (THER), drug indications (INDI), patient outcomes information (OUCT), and reported sources (RPSR). A relational database was established to link these seven datasets through the specific identification numbers present in each FAERS report. For the present analysis, FAERS data from the first quarter of 2004 to the fourth quarter of 2024 were utilized. Deduplication was carried out following FDA guidelines. When PRIMARYIDs were identical, the latest FDA_DT was selected. In cases where both FDA_DT and CASEID were the same, the higher PRIMARYID was chosen.

### Identification of AEs and drugs

Adverse events in the FAERS database were coded with preferred terms (PTs) using the Medical Dictionary for Regulatory Activities, version 27.1 (MedDRA 27.1). The PT ‘DI’ (code: 10012599) was employed to identify the targeted AE. Drugs associated with reported AEs were categorized into four roles: primary suspect (PS), secondary suspect (SS), concomitant (C), and interacting (I). Only cases where the drug role was coded as PS were selected for analysis. Drug names were standardized based on the DrugBank database (https://go.drugbank.com/) and the MeSH Database (https://www.ncbi.nlm.nih.gov/mesh/?term=). The Anatomical Therapeutic Chemical (ATC) classification system, which is developed and regularly updated by the WHO Collaborating Centre for Drug Statistics Methodology, was adopted for drug classification.

### Statistical analysis

Descriptive statistics were used to characterize AE reports related to DI, including gender, age, weight, outcomes, reporter occupation, country of origin, and reporting year. To identify potential agents associated with DI, disproportionality analyses were conducted using the reporting odds ratio (ROR) and the Bayesian confidence propagation neural network (BCPNN) (12). The formulas for ROR and BCPNN are presented in Table 1. To enhance the reliability of the drug-AE correlation analysis, a positive signal was considered to exist only when the criteria for both algorithms were concurrently met: the lower 95% confidence interval (CI) of ROR > 1, cases number ≥3, and the lower limit of the information component (IC_025_) for BCPNN > 0. In addition, signal strength was stratified based on IC_025_, with an IC_025_ value between 0 and 1.5 categorized as a weak signal, a value between 1.5 and 3 designated as a medium signal, and a value greater than 3 classified as a strong signal (12, 13). Furthermore, the onset time was described using the median and interquartile range (IQR) and calculated as the interval between the AE occurrence date (EVENT_DT) and the agent initiation date (START_DT). It is imperative to note that entries containing incomplete, missing, or incorrectly formatted date information were excluded from the onset time calculation. All statistical analyses and data visualization were performed using R software version 4.4.2 and Microsoft Excel 2019.

**Table 1:** Algorithms employed for signal detection.

Algorithms	Equation	Criteria
ROR	ROR = *ad*/*b*/*c*	• Lower limit of 95% • CI > 1, *N* ≥ 3
	95% CI = e^ln(ROR) ± 1.96(1/*a* + 1/*b* + 1/*c* + 1/*d*)^0.5^	
BCPNN	IC = log_2_*a*(*a* + *b* + *c* + *d*)(*a* + *c*)(*a* + *b*)	• IC_025_ > 0
	$\gamma \;=\;\gamma_{\text{ij}}\frac{\left(N+\alpha \right)\left(N+\beta \right)}{\left(a+b+\alpha_{i}\right)\left(a+c+\beta_{j}\right)}$	
	$E\left(\text{IC}\right)=\log_{2}\left(\frac{\left(a+\gamma_{\text{ij}}\right)\left(N+\alpha \right)\left(N+\beta \right)}{\left(N+\gamma \right)\left(a+b+\alpha_{i}\right)\left(a+c+\beta_{j}\right)}\right)$	
	$V\left(\text{IC}\right)=\frac{1}{{\left(\log2\right)}^{2}}\left[\frac{N-a+\gamma -\gamma_{\text{ij}}}{\left(a+\gamma_{\text{ij}}\right)\left(1+N+\gamma \right)}\;+\frac{N-a-b+\alpha -\alpha_{i}}{\left(a+b+\alpha_{i}\right)\left(1+N+\alpha \right)}+\frac{N-a-c+\beta -\beta_{j}}{\left(a+c+\beta_{j}\right)\left(1+N+\beta \right)}\right]$	
	${\text{IC}}_{025}=\;E\left(\text{IC}\right)-2\sqrt{V\left(\text{IC}\right)}$	

*a*: number of diabetes insipidus cases related to the target drug; *b*: number of other adverse event cases related to the target drug; *c*: number of diabetes insipidus cases related to background drugs; *d*: number of other adverse event cases related to the background drug; *N*: the number of total cases.

CI, confidence interval; N, the number of reports; IC, information component; IC_025_, the lower 95% CI for the IC; E(IC), the IC expectations; V(IC), the variance of IC; BCPNN, Bayesian confidence propagation neural network; ROR, reporting odds ratio.

## Results

### Descriptive analysis

**Figure 1:**
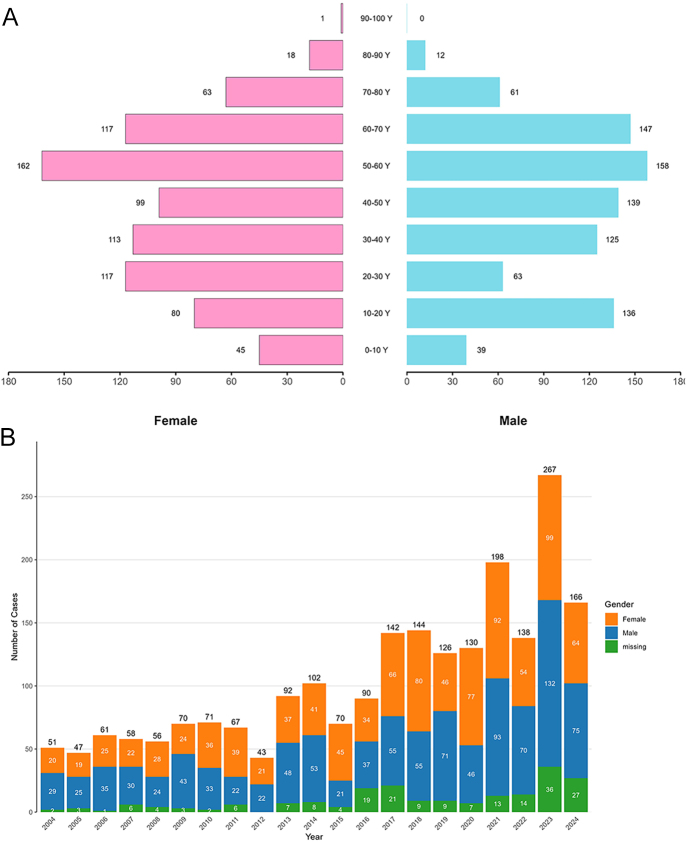
Characteristics of drug-related DI reports from the FDA Adverse Event Reporting System database. (A) Population pyramid of subjects with drug-induced DI categorized by gender and age. (B) Bar chart depicting the annual reporting frequencies of drug-induced DI, stratified by gender.

**Figure 2:**
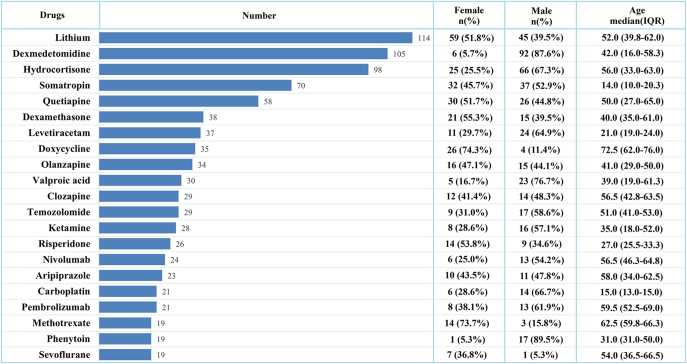
Top 21 drugs associated with the highest number of reported cases of DI.

From the first quarter of 2004 to the third quarter of 2024, the FAERS database, after deduplication, documented 18,627,667 cases, of which 2,189 were reported as cases of DI (Supplementary Fig. S1 (see section on Supplementary materials given at the end of the article)). The median age of these patients was 47.0 years (IQR 27.0–60.0). Females constituted 44.3% of the cases, and males accounted for 46.6% (Table 2). The patients’ ages primarily fell within the range of 50–70 years for both genders (Fig. 1A). There has been an increasing epidemiological trend in reported cases of drug-associated DI, reaching its peak in 2023 with 267 cases (Fig. 1B). Among these drug-associated DI cases, 266 (12.2%) reported outcomes were death, 135 (6.2%) were life-threatening, and 760 (34.7%) required hospitalization. 80.4% of the cases were submitted by healthcare professionals, with physicians being the largest contributors at 805 (36.8%). The majority of reports originated from the United States (789, 36.0%), followed by Japan (276, 12.6%) and France (188, 8.6%). Lithium was the most frequently reported drug (114 cases) with a median age of 52.0 years (IQR 39.8–62.0), followed by dexmedetomidine (105 cases) with a median age of 42.0 years (IQR 16.0–58.3) (Fig. 2).

**Table 2:** Demographics and characteristics of patients with drug-associated diabetes insipidus reported in the FAERS database.

Characteristics	Values
Number of total cases	2,189
Gender	
Female	969 (44.3%)
Male	1,019 (46.6%)
Unknown	201 (9.2%)
Age (years)	
<18	253 (11.6%)
18–64	1,185 (54.1%)
≥65	286 (13.1%)
Unknown	465 (21.2%)
Median (IQR)	47.0 (27.0–60.0)
Weight (kg)	
<80	307 (14.0%)
≥80	219 (10.0%)
Unknown	1,663 (76.0%)
Median (IQR)	74.5 (57.0–88.0)
Outcomes	
Death	266 (12.2%)
Life-threatening	135 (6.2%)
Hospitalization – initial or prolonged	760 (34.7%)
Disability	20 (0.9%)
Congenital anomaly	5 (0.2%)
Required intervention	7 (0.3%)
Other serious	973 (44.4%)
Unknown	23 (1.1%)
Reporters	
Physician	805 (36.8%)
Pharmacist	166 (7.6%)
Other health-professional	787 (36.0%)
Consumer	293 (13.4%)
Lawyer	8 (0.4%)
Unknown	130 (5.9%)
TOP 5 report countries	
United States	789 (36.0%)
Japan	276 (12.6%)
France	188 (8.6%)
Canada	153 (7.0%)
United Kingdom	129 (5.9%)

IQR, interquartile range.

### Disproportionality analysis

**Figure 3:**
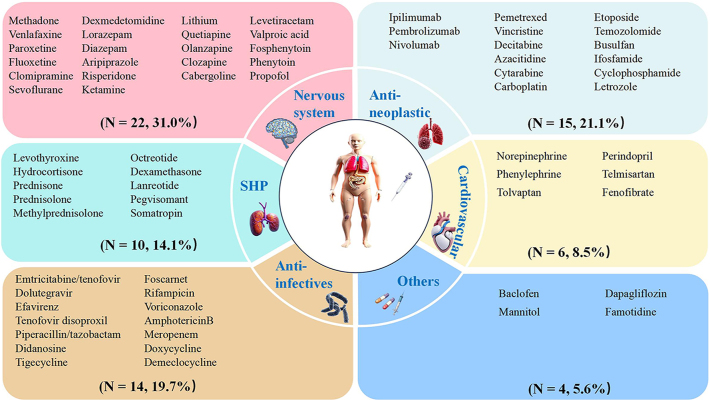
Classification of medications associated with DI utilizing the Anatomical Therapeutic Chemical classification system.

**Figure 4:**
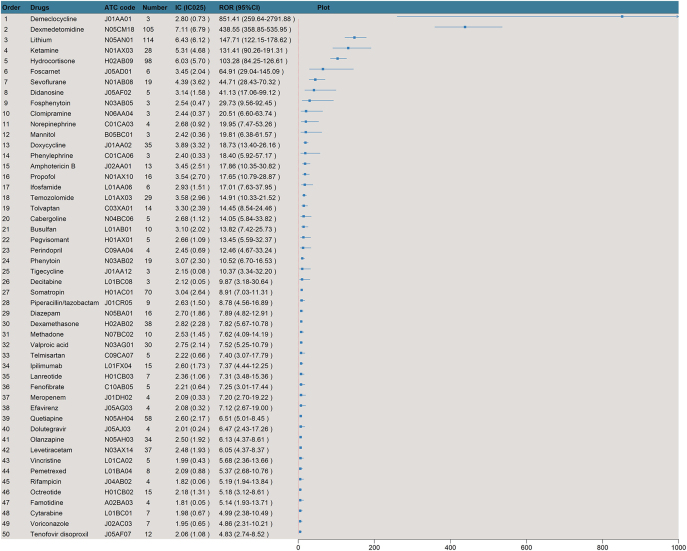
Forest plot of the top 50 drugs ranked by the reporting odds ratio value. ATC, Anatomical Therapeutic Chemical classification; CI, confidence interval; IC, information component; IC_025,_ the lower 95% CI for the IC; ROR, reporting odds ratio.

**Figure 5:**
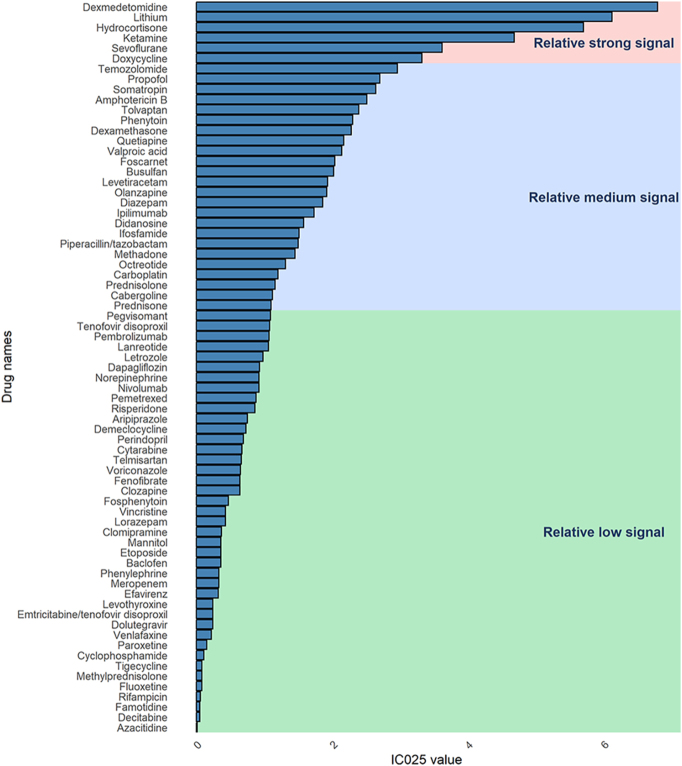
Stratification of drug signal strength based on IC_025_. IC_025_, the lower 95% CI for the information component.

A total of 71 drugs were identified as potentially associated with DI (Supplementary Table S1). The largest number of these drugs belonged to nervous system agents, with 22 drugs (31.0%). This was followed by antineoplastic and immunomodulating agents, which had 15 drugs (21.1%), anti-infectives for systemic use with 14 drugs (19.7%), systemic hormonal preparations with 10 drugs (14.1%), and cardiovascular system drugs with 6 drugs (8.5%) (Fig. 3). Demeclocycline was found to be the drug with the ROR for DI, having an ROR of 851.41 (95% CI: 259.64–2,791.88). Subsequently, dexmedetomidine ranked next with an ROR of 438.55 (95% CI: 358.85–535.95), and lithium with an ROR of 147.71 (95% CI: 122.15–178.62) (Fig. 4). Among the 71 potential drugs, they were categorized into high, medium, and low signal categories according to the BCPNN. Six drugs (8.5%) were classified as strong signals, 18 drugs (25.4%) as medium signals, and 48 drugs (66.2%) as low signals (Fig. 5). The drugs with strong signals included dexmedetomidine (IC_025_ = 6.79), lithium (IC_025_ = 6.12), hydrocortisone (IC_025_ = 5.70), ketamine (IC_025_ = 4.68), sevoflurane (IC_025_ = 3.62), and doxycycline (IC_025_ = 3.32).

### Time-to-onset analysis

Among the 71 drugs identified as having a positive signal associated with DI, complete onset-time data were available for 46 drugs (Table 3). Our analysis indicated that dolutegravir, cyclophosphamide, tolvaptan, and dexmedetomidine had an immediate onset, with a median onset time of 0 days. Conversely, the antipsychotics clozapine and risperidone displayed the latest onsets, with median onset times of 1,776.5 days (IQR 326.3–3,482.5) and 1,699.0 days (IQR 920.5–2,477.5), respectively. Notably, antineoplastic agents presented heterogeneous onset time patterns. ICIs, such as nivolumab (median 78.5 days, IQR 21.8–138.3), pembrolizumab (median 40.0 days, IQR 19.0–136.0), and ipilimumab (median 45.5 days, IQR 32.0–54.0), demonstrated intermediate latencies. In contrast, cytotoxic agents, especially cyclophosphamide (median 0 days, IQR 0–0) and pemetrexed (median 4.5 days, IQR 2.3–6.8), showed rapid onsets.

**Table 3:** Onset time for the signal-positive drugs (*n* = 46).

Drugs	Category	Available cases (*n*)	TTO (days)
			Median (IQR)
Dapagliflozin	Alimentary tract & metabolism	4	5.5 (3–8.5)
Famotidine	Alimentary tract & metabolism	1	1.0 (1.0–1.0)
Amphotericin B	Anti-infectives for systemic use	5	4.0 (3.0–6.0)
Tenofovir disoproxil	Anti-infectives for systemic use	4	373.0 (327.3–645.5)
Piperacillin/tazobactam	Anti-infectives for systemic use	4	5.0 (3.8–6.0)
Emtricitabine/tenofovir disoproxil	Anti-infectives for systemic use	2	108.0 (86.5–129.5)
Voriconazole	Anti-infectives for systemic use	3	7.0 (6.0–9.0)
Foscarnet	Anti-infectives for systemic use	1	9.0 (9.0–9.0)
Dolutegravir	Anti-infectives for systemic use	2	0 (0–0)
Efavirenz	Anti-infectives for systemic use	1	6.0 (6.0–6.0)
Meropenem	Anti-infectives for systemic use	1	5.0 (5.0–5.0)
Tigecycline	Anti-infectives for systemic use	1	9.0 (9.0–9.0)
Temozolomide	AAIA	4	41.5 (27.0–52.8)
Nivolumab	AAIA	8	78.5 (21.8–138.3)
Pembrolizumab	AAIA	9	40.0 (19.0–136.0)
Ipilimumab	AAIA	4	45.5 (32.0–54.0)
Letrozole	AAIA	2	147.0 (87.5–206.5)
Busulfan	AAIA	5	25.0 (20.0–36.0)
Carboplatin	AAIA	1	6.0 (6.0–6.0)
Cyclophosphamide	AAIA	2	0 (0–0)
Pemetrexed	AAIA	2	4.5 (2.3–6.8)
Cytarabine	AAIA	1	2.0 (2.0–2.0)
Azacitidine	AAIA	3	6.0 (3.0–58.0)
Etoposide	AAIA	1	18.0 (18.0–18.0)
Ifosfamide	AAIA	1	1.0 (1.0–1.0)
Vincristine	AAIA	2	141.0 (70.5–211.5)
Mannitol	Blood and blood forming organs	2	2.0 (1.5–2.5)
Tolvaptan	Cardiovascular system	12	0 (0–3.5)
Norepinephrine	Cardiovascular system	2	3.0 (3.0–3.0)
Lithium	Nervous system	16	226.5 (55.5–1,338.8)
Dexmedetomidine	Nervous system	5	0 (0–1.0)
Quetiapine	Nervous system	12	307.5 (30.8–410.5)
Levetiracetam	Nervous system	2	45.5 (22.8–68.3)
Olanzapine	Nervous system	7	31.0 (3.0–1,434.5)
Valproic acid	Nervous system	5	60.0 (37–1,310)
Clozapine	Nervous system	8	1,776.5 (326.3–3,482.5)
Risperidone	Nervous system	2	1,699.0 (920.5–2,477.5)
Aripiprazole	Nervous system	5	1.0 (1.0–3.0)
Sevoflurane	Nervous system	8	2.0 (2.0–5.0)
Paroxetine	Nervous system	1	790.0 (790.0–790.0)
Hydrocortisone	Systemic hormonal preparations	4	77.5 (2.5–205.5)
Somatropin	Systemic hormonal preparations	15	898.0 (253.0–1,909.0)
Dexamethasone	Systemic hormonal preparations	2	2.0 (1.0–3.0)
Octreotide	Systemic hormonal preparations	2	1,449.0 (1,077.5–1,820.5)
Lanreotide	Systemic hormonal preparations	2	274.0 (151.5–396.5)
Pegvisomant	Systemic hormonal preparations	1	37.0 (37.0–37.0)

AAIA, antineoplastic and immunomodulating agents; TTO, time-to-onset; IQR, interquartile range.

## Discussion

To the best of our knowledge, this study is the first to undertake a comprehensive pharmacovigilance analysis of DI using real-world data from the FAERS database. DI, a rare endocrine disorder, can manifest at any age and is equally prevalent between genders, which is consistent with our demographic findings (2). During the 20-year review period from Q1 2004 to Q4 2024, FAERS documented only 2,189 cases of DI, with a median age of 47.0 years (IQR 27.0–60.0). The gender distribution of cases was nearly equal, with females accounting for 969 cases (44.3%) and males reporting 1,019 cases (46.6%). However, potential gender disparities were observed when examining DI cases associated with specific drugs. For instance, among cases associated with dexmedetomidine, 92 (87.6%) were male, and for phenytoin-related cases, 17 (89.5%) were male. Conversely, for cases associated with methotrexate, 14 (73.7%) were female. Moreover, an upward trend in the annual reporting of cases of drug-induced DI was observed, which may be attributed to enhanced clinical recognition and the increased utilization of novel high-risk drugs.

A total of 71 drugs, belonging to 8 first-level anatomical main groups of the ATC classification, were identified as potentially associated with DI in the present study. Agents of the nervous system constituted the largest proportion of DI-associated drugs (31.0%), including anesthetics, hypnotics, sedatives, antiepileptics, anti-parkinsonian agents, antipsychotics, anxiolytics, and psychostimulants. Lithium (*n* = 114, IC_025_ = 6.12) was the most frequently reported and the second–strongest association signal. Lithium, a well-established cause of NDI, exerts its effects through the disruption of renal collecting duct function. This disruption is mediated by epithelial sodium channel-mediated cellular uptake, glycogen synthase kinase 3 inhibition, and subsequent downregulation or mistrafficking of aquaporin-2 (AQP2) (14, 15). The occurrence of lithium-induced NDI has been shown to be dependent on the cumulative dose and duration of treatment (16). Our analysis revealed a median onset time of 226.5 days (IQR 55.5–1,338.8) for lithium-associated DI, consistent with the characteristic temporal pattern of lithium’s cumulative nephrotoxicity. In addition to lithium, antipsychotic drugs such as clozapine, olanzapine, quetiapine, risperidone, and aripiprazole also exhibited positive association signals. While there are existing reports indicating DI associated with clozapine, olanzapine, quetiapine, and risperidone (8, 17, 18, 19), no published data on aripiprazole-induced DI are available to date.

Dexmedetomidine, a widely used sedative in the intensive care unit (ICU) and during surgical procedures, was identified as the second most frequently reported drug and showed the strongest association with DI in our analysis (*n* = 105, IC_025_ = 6.79). Although DI is not currently listed as an adverse effect in the label of dexmedetomidine, several case reports have emerged suggesting a possible association between dexmedetomidine use and the development of DI (20, 21, 22). As a highly selective α2-adrenergic receptor agonist, dexmedetomidine may induce central DI by activating α2 receptors in the central nervous system, thereby inhibiting the release of AVP from the hypothalamus or posterior pituitary (23). In our analysis, the onset times for dexmedetomidine-associated DI were documented in five cases, with three cases developing DI on the first day of dexmedetomidine administration and two additional cases on the day after the first dose, consistent with case reports. Vani *et al.* (22) reported that a 32-year-old male patient admitted to the surgical ICU with 50% total body surface area burns developed symptoms of DI three hours after initiation of a dexmedetomidine infusion. In addition, a 55-year-old male patient undergoing coronary artery bypass graft surgery experienced symptoms of DI during surgery after receiving dexmedetomidine (21). These findings underscore the importance of closely monitoring urine output and electrolyte balance in patients at the initiation of dexmedetomidine therapy.

The results of our disproportionality analysis indicated that 15 antineoplastic drugs, including chemotherapeutic agents, ICIs, and endocrine therapy drugs, are potentially associated with DI. Previous studies have suggested that chemotherapy-related DI is predominantly of the nephrogenic subtype, with case reports implicating agents such as pemetrexed, cyclophosphamide, and ifosfamide (24, 25, 26). It has been hypothesized that pemetrexed directly interferes with the function or expression of AQP2 by targeting the folate transport system in the collecting ducts, resulting in NDI (24). Ifosfamide and its metabolites exhibit direct toxicity to renal tubular epithelial cells, reducing the sensitivity of the renal tubules to AVP or even causing complete resistance, thus triggering NDI (26). However, temozolomide, an oral DNA-alkylating chemotherapeutic agent, has been associated with impaired release of AVP and oxytocin, leading to CDI (27).

CDI is an extremely rare but increasingly recognized immune-related adverse event during ICI therapy (9, 28, 29). Following the relief of T-cell inhibition by ICIs, there is a possibility that they may trigger an autoimmune attack against the hypothalamic–neurohypophyseal axis, resulting in insufficient secretion of AVP and thus inducing CDI (9, 29). Anti-cytotoxic T-lymphocyte-associated antigen 4 agents may mainly affect anterior pituitary cells, while programmed cell death protein 1/programmed death-ligand 1 inhibitors appear to be expressed in hypothalamic neurons. Furthermore, our analysis found that the median onset time of ICI-induced CDI was 40.0–78.5 days, which appeared to be marginally earlier than that documented in sporadic case reports. Barnabei *et al.* conducted a review of 11 cases of ICI-induced CDI from case reports and showed that the median induction time of CDI in these cases was 98.0 days (IQR 41.3–142.8) (9). Conversely, our analysis suggested that the onset of ICI-related DI occurs later than that of chemotherapy-related DI.

An unexpected positive signal was observed for letrozole, with an ROR of 4.68 (95% CI: 2.59–8.47) and an IC_025_ of 0.98 in our analysis. Letrozole, an aromatase inhibitor, functions by reducing estrogen levels in the body through the inhibition of estrogen synthesis and is primarily indicated for the treatment of breast cancer (30). The FAERS database recorded 11 cases of DI associated with letrozole, with a median onset time of 147.0 days (IQR 87.5–206.5). To date, no clinical reports have directly linked letrozole to DI. However, a mechanistic hypothesis can be proposed based on its pharmacological profile. Evidence from previous studies indicates that estrogen modulates AVP expression and secretion through estrogen receptors (ERα/ERβ) (31, 32). Thus, the profound estrogen depletion resulting from prolonged letrozole use may disrupt AVP homeostasis, potentially increasing DI risk. Further studies are warranted to explore this association and elucidate the underlying pathophysiology.

Our analysis revealed that antimicrobial agents, including antibacterial, antifungal, and antiviral drugs, may contribute to DI development. Notably, doxycycline demonstrated a strong signal (IC_025_ = 3.32), though evidence supporting this association remains limited. To date, only a single case report has described DI onset in a patient with chronic driveline infections following prolonged doxycycline therapy (33). Demeclocycline, another member of the tetracycline family, is a well-documented inducer of NDI. The proposed mechanism involves demeclocycline’s entry into the renal collecting duct through transporter channels, human organic anion transporter 1 and human organic anion transporter 3, located on the basolateral membrane, thereby inhibiting AVP-induced water flow (5). Our analysis identified a novel signal for tigecycline (ROR = 10.37, 95% CI: 3.34–32.2; IC_025_ = 0.08), yet no clinical reports of tigecycline-associated DI were retrievable from the existing literature. These findings highlight the need for further investigation into the association between these antimicrobials and DI.

Octreotide and lanreotide are synthetic somatostatin analogs that mimic the effects of natural somatostatin (34, 35). These medications are primarily employed for the regulation of hormone secretion and the inhibition of tumor growth. Our findings indicated that both octreotide (ROR = 5.18, 95% CI: 3.12–8.61, IC_025_ = 1.31) and lanreotide (ROR = 7.31, 95% CI: 3.48–15.36, IC_025_ = 1.06) may induce DI. The DI associated with these analogs is classified as CDI. A case report documented a favorable response to desmopressin treatment in a patient with DI following the concomitant use of octreotide and lanreotide (36). These somatostatin analogs directly influence the hypothalamic–posterior pituitary axis by activating somatostatin receptors in the posterior pituitary, thereby inhibiting AVP synthesis and release (36). Given the overlapping distribution of somatostatin and AVP in the posterior pituitary, these analogs might indirectly reduce AVP secretion by altering the excitability of glutamatergic neurons or enhancing the inhibitory effects of gamma-aminobutyric acid (36).

Our analysis also identified several novel signals not previously described, such as dapagliflozin, famotidine, pegvisomant, perindopril, and telmisartan. Dapagliflozin, by inhibiting sodium–glucose cotransporter 2 in the renal tubules, reduces the reabsorption of glucose and sodium, leading to osmotic diuresis (37). The dehydration induced by dapagliflozin may activate AVP secretion through osmoreceptors and volume sensors (38). However, sustained elevation of AVP may lead to downregulation or desensitization of renal AVP receptors, thereby reducing the tubular responsiveness to AVP and decreasing the capacity for urine concentration (39). Whether this mechanism is responsible for the development of DI induced by dapagliflozin remains to be determined and requires further experimental investigation.

The present study is subject to certain limitations. First, as a spontaneous reporting system, the FAERS database is inherently susceptible to reporting biases, despite its global coverage of adverse events. The absence of comprehensive patient case information, including but not limited to age, gender, and dosage, can lead to inaccuracies and potential data omissions, thereby risking under- or overestimation of specific AEs. Second, while disproportionality analysis is useful for identifying potential drug–event associations, the absence of clinical adjudication in FAERS data limits the confirmation of causality. The temporal relationships reported may be coincidental, particularly for rare events such as DI, and could be influenced by concurrent conditions (e.g., electrolyte imbalances) or unmeasured confounders. Third, it is important to note that disproportionality analysis is a tool for generating hypotheses, not for testing. While it can indicate potential drug safety concerns, it lacks the ability to establish causality between drugs and adverse events. To improve the accuracy of AE identification, future efforts should integrate data from multiple sources, including drug labels, the published literature, and clinical guidelines, thereby enabling a more robust, evidence-based assessment.

## Conclusion

This study systematically evaluated the epidemiological characteristics and potential causative medications of drug-induced DI through analysis of the FAERS database. The disproportionality analysis identified a range of drugs associated with DI, with the largest percentage of drugs being nervous system agents, antineoplastic drugs, and antimicrobials. The potential mechanisms primarily involve drug-induced interference with AVP secretion from the hypothalamic–pituitary axis or a diminished renal response to AVP. Lithium and dexmedetomidine emerged as prototypical agents for nephrogenic NDI and CDI, respectively, demonstrating the strongest signal intensities. Novel potential associations between DI and medications such as aripiprazole, letrozole, tigecycline, and dapagliflozin were first reported in this investigation. Future studies should integrate multisource data to validate these signals and employ experimental approaches to elucidate the molecular mechanisms underlying these newly identified associations, thereby optimizing pharmacovigilance strategies and DI management protocols.

## Supplementary materials



## Declaration of interest

The authors declare that there is no conflict of interest that could be perceived as prejudicing the impartiality of the work reported.

## Funding

This work did not receive any specific grant from any funding agency in the public, commercial, or not-for-profit sector.

## Author contribution statement

Contributors NS conceived and designed the study. HL, YW, and LL performed the data extraction and data analyses. HL drafted the manuscript. YW, LL, and NS revised the manuscript. HL is the guarantor of the manuscript and accepts full responsibility for the work. All authors have read and approved the version of the manuscript submitted for publication.

## Data availability

The datasets used and/or analyzed during the current study are available from the corresponding author on reasonable request.

## Ethics approval and consent to participate

No institutional ethics approval was required because this study utilized anonymized data from an open-access database.
