# Supplementary material for: Real-world pharmacovigilance study of drug-induced diabetes insipidus from the FAERS database
Source: Endocr Connect. 2026 Jan 2;15(1):e250734. doi: 10.1530/EC-25-0734 (PMC12766370; doi:10.1530/EC-25-0734)
Supplement: Supplementary file 1 [file supplementary_materials.pdf]

Supplementary Information

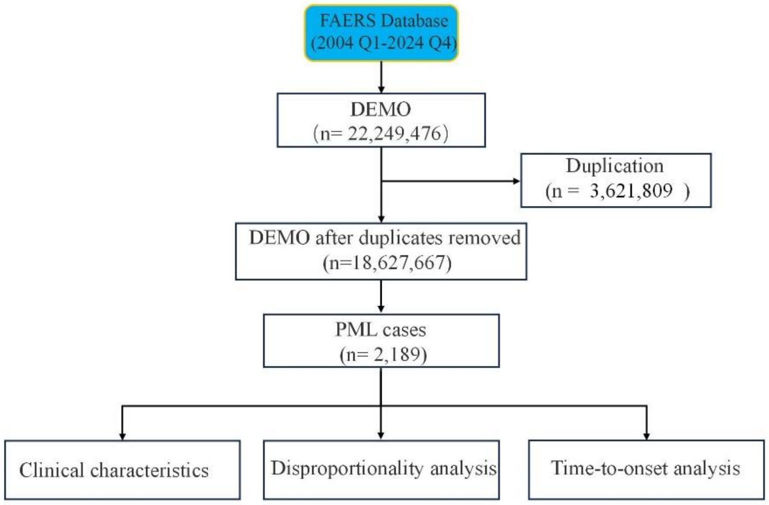

Figure S1. Flow Diagram of Screening Reports Associated with Diabetes Insipidus from the FAERS Database

Table S1. Drugs with Positive Signals Associated with Diabetes Insipidus

Classification	Drugs	ATC code	Cases	ROR (95% CI)	IC (IC025)
Alimentary tract and metabolism	Famotidine	A02BA03	4	5.14 (1.93-13.71)	1.81 (0.05)
	Dapagliflozin	A10BK01	13	4.08 (2.37-7.04)	1.87 (0.93)
Blood and blood forming organs	Mannitol	B05BC01	3	19.81 (6.38-61.57)	2.42 (0.36)
Cardiovascular system	Norepinephrine	C01CA03	4	19.95 (7.47-53.26)	2.68 (0.92)
	Phenylephrine	C01CA06	3	18.4 (5.92-57.17)	2.40 (0.33)
	Tolvaptan	C03XA01	14	14.45 (8.54-24.46)	3.30 (2.39)
	Perindopril	C09AA04	4	12.46 (4.67-33.24)	2.45 (0.69)
	Telmisartan	C09CA07	5	7.4 (3.07-17.79)	2.22 (0.66)
	Fenofibrate	C10AB05	5	7.25 (3.01-17.44)	2.21 (0.64)
Systemic hormonal preparations	Somatropin	H01AC01	70	8.91 (7.03-11.31)	3.04 (2.64)
	Pegvisomant	H01AX01	5	13.45 (5.59-32.37)	2.66 (1.09)
	Octreotide	H01CB02	15	5.18 (3.12-8.61)	2.18 (1.31)
	Lanreotide	H01CB03	7	7.31 (3.48-15.36)	2.36 (1.06)
	Dexamethasone	H02AB02	38	7.82 (5.67-10.78)	2.82 (2.28)
	Methylprednisolone	H02AB04	8	2.71 (1.35-5.43)	1.30 (0.08)
	Prednisolone	H02AB06	17	4.35 (2.7-7.01)	1.98 (1.16)
	Prednisone	H02AB07	17	4.12 (2.56-6.65)	1.91 (1.10)
	Hydrocortisone	H02AB09	98	103.28 (84.25-126.61)	6.03 (5.70)
	Levothyroxine	H03AA01	14	2.32 (1.37-3.93)	1.15 (0.24)
Anti-infectives for systemic use	Demeclocycline	J01AA01	3	851.41 (259.64-2791.88)	2.80 (0.73)
	Doxycycline	J01AA02	35	18.73 (13.4-26.16)	3.89 (3.32)

	Tigecycline	J01AA12	3	10.37 (3.34-32.2)	2.15 (0.08)
	Piperacillin/tazobactam	J01CR05	9	8.78 (4.56-16.89)	2.63 (1.50)
	Meropenem	J01DH02	4	7.2 (2.7-19.22)	2.09 (0.33)
	Amphotericin B	J02AA01	13	17.86 (10.35-30.82)	3.45 (2.51)
	Voriconazole	J02AC03	7	4.86 (2.31-10.21)	1.95 (0.65)
	Rifampicin	J04AB02	4	5.19 (1.94-13.84)	1.82 (0.06)
	Foscarnet	J05AD01	6	64.91 (29.04-145.09)	3.45 (2.04)
	Didanosine	J05AF02	5	41.13 (17.06-99.12)	3.14 (1.58)
	Tenofovir disoproxil	J05AF07	12	4.83 (2.74-8.52)	2.06 (1.08)
	Efavirenz	J05AG03	4	7.12 (2.67-19)	2.08 (0.32)
	Dolutegravir	J05AJ03	4	6.47 (2.43-17.26)	2.01 (0.24)
	Emtricitabine/tenofovir disoproxil	J05AR03	8	3.09 (1.54-6.18)	1.46 (0.24)
Antineoplastic and immunomodulating agents	Cyclophosphamide	L01AA01	9	2.57 (1.34-4.95)	1.24 (0.11)
	Ifosfamide	L01AA06	6	17.01 (7.63-37.95)	2.93 (1.51)
	Busulfan	L01AB01	10	13.82 (7.42-25.73)	3.10 (2.02)
	Temozolomide	L01AX03	29	14.91 (10.33-21.52)	3.58 (2.96)
	Pemetrexed	L01BA04	8	5.37 (2.68-10.76)	2.09 (0.88)
	Cytarabine	L01BC01	7	4.99 (2.38-10.49)	1.98 (0.67)
	Azacitidine	L01BC07	6	3.13 (1.4-6.97)	1.42 (0.01)
	Decitabine	L01BC08	3	9.87 (3.18-30.64)	2.12 (0.05)
	Vincristine	L01CA02	5	5.68 (2.36-13.66)	1.99 (0.43)
	Etoposide	L01CB01	6	4.31 (1.93-9.6)	1.78 (0.36)
	Nivolumab	L01FF01	24	3.21 (2.15-4.8)	1.61 (0.92)

	Pembrolizumab	L01FF02	21	3.74 (2.43-5.75)	1.80 (1.07)
	Ipilimumab	L01FX04	15	7.37 (4.44-12.25)	2.60 (1.73)
	Carboplatin	L01XA02	21	4.13 (2.69-6.35)	1.93 (1.20)
	Letrozole	L02BG04	11	4.68 (2.59-8.47)	2.01 (0.98)
Musculo-skeletal system	Baclofen	M03BX01	10	2.97 (1.59-5.52)	1.44 (0.36)
Nervous system	Sevoflurane	N01AB08	19	44.71 (28.43-70.32)	4.39 (3.62)
	Ketamine	N01AX03	28	131.41 (90.26-191.31)	5.31 (4.68)
	Propofol	N01AX10	16	17.65 (10.79-28.87)	3.54 (2.70)
	Phenytoin	N03AB02	19	10.52 (6.7-16.53)	3.07 (2.30)
	Fosphenytoin	N03AB05	3	29.73 (9.56-92.45)	2.54 (0.47)
	Valproic acid	N03AG01	30	7.52 (5.25-10.79)	2.75 (2.14)
	Levetiracetam	N03AX14	37	6.05 (4.37-8.37)	2.48 (1.93)
	Cabergoline	N04BC06	5	14.05 (5.84-33.82)	2.68 (1.12)
	Clozapine	N05AH02	29	2.48 (1.72-3.58)	1.26 (0.64)
	Olanzapine	N05AH03	34	6.13 (4.37-8.61)	2.50 (1.92)
	Quetiapine	N05AH04	58	6.51 (5.01-8.45)	2.60 (2.17)
	Lithium	N05AN01	114	147.71 (122.15-178.62)	6.43 (6.12)
	Risperidone	N05AX08	26	2.99 (2.03-4.4)	1.51 (0.86)
	Aripiprazole	N05AX12	23	2.85 (1.89-4.29)	1.44 (0.75)
	Diazepam	N05BA01	16	7.89 (4.82-12.91)	2.70 (1.86)
	Lorazepam	N05BA06	7	3.98 (1.9-8.37)	1.73 (0.43)
	Dexmedetomidine	N05CM18	105	438.55 (358.85-535.95)	7.11 (6.79)
	Clomipramine	N06AA04	3	20.51 (6.6-63.74)	2.44 (0.37)
	Fluoxetine	N06AB03	8	2.7 (1.35-5.41)	1.29 (0.08)

	Paroxetine	N06AB05	11	2.4 (1.33-4.34)	1.17 (0.15)
	Venlafaxine	N06AX16	12	2.44 (1.39-4.31)	1.20 (0.22)
	Methadone	N07BC02	10	7.62 (4.09-14.19)	2.53 (1.45)
